# Reactive Oxygen Species Production in Peripheral Blood Neutrophils of Obstructive Sleep Apnea Patients

**DOI:** 10.1155/2013/421763

**Published:** 2013-05-12

**Authors:** Guoda Pilkauskaite, Skaidrius Miliauskas, Raimundas Sakalauskas

**Affiliations:** Department of Pulmonology and Immunology, Medical Academy, Lithuanian University of Health Sciences, Eiveniu 2, 50009 Kaunas, Lithuania

## Abstract

Obstructive sleep apnea (OSA) as well as obesity is associated with increased production of reactive oxygen species (ROS). Neutrophils produce great amounts of ROS. The aim was to evaluate peripheral blood neutrophils ROS production in men with OSA and to establish relations with disease severity and obesity. *Methods*. Forty-six men with OSA and 10 controls were investigated. OSA was confirmed by polysomnography (PSG), when apnea/hypopnea index was >5/h. Body mass index (BMI) was evaluated. Neutrophils were isolated from peripheral blood in the morning after PSG. Dihydrorhodamine-123 was used for ROS detection. Data is presented as median (25th and 75th percentiles). All subjects were divided into four groups: nonobese mild-to-moderate OSA, obese mild-to-moderate OSA, nonobese severe OSA, and obese severe OSA. *Results*. Neutrophil ROS production was higher in nonobese severe OSA group compared to nonobese mild-to-moderate OSA (mean fluorescence intensity (MFI) 213.4 (89.0–238.9) versus 44.5 (20.5–58.4), *P* < 0.05). In obese patient groups, ROS production was more increased in severe OSA compared to mild-to-moderate OSA group (MFI 74.5 (47.9–182.4) versus 31.0 (14.8–53.8), *P* < 0.05). It did not differ in the groups with different BMI and the same severity of OSA. *Conclusion*. Increased neutrophil ROS production was related to more severe OSA but not obesity.

## 1. Introduction

Obstructive sleep apnea (OSA) is characterized by repetitive pauses of breathing caused by partial or complete collapses of upper airways during sleep. The prevalence of this sleep-related breathing disorder is reported to be about 4% in middle-aged men [1]. OSA is associated with daytime sleepiness, impaired quality of life, arterial hypertension, obesity, and other components of metabolic syndrome as well as coronary artery disease [2–4]. It is an independent risk factor for stroke and death [4–6].

In OSA, pauses of breathing are followed by decreased oxygen saturation and arousals during sleep that result in hypoxia/reoxygenation circles. This pattern is also called intermittent hypoxia. Intermittent hypoxia is considered to be analogous to the conditions of ischemia and reperfusion injury but not as aggressive as it has been demonstrated in ischemic heart disease [7]. 

Hypoxia is a well-explained stimulus in activation of various cells in peripheral blood that leads to increased burst of reactive oxygen species (ROS) [8]. ROS molecules produced in response of intermittent hypoxia initiate the cascade of inflammatory pathways resulting in overexpression of pro-inflammatory cytokines and adhesion molecules. Oxidizing radicals and proteolytic enzymes affect endothelial function during accumulation of leucocytes and platelets on the endothelium and interaction with the vascular wall. This leads to endothelial dysfunction—a subclinical condition of atherosclerosis promoting formation of cardiovascular diseases [9]. 

Studies of cell culture, animal models, and humans have demonstrated that increased ROS formation and oxidative stress are the results of OSA. However, much of the data is based on indirect evidence, mostly measuring circulating markers of oxidative stress [10, 11]. Activation and overexpression of ROS have been demonstrated in various blood cells as well. Lymphocytes and monocytes have been studied precisely in OSA showing the ability to initiate inflammatory cascade by overproduction of ROS while exposed to intermittent hypoxia [12, 13]. Neutrophils should play an important role in OSA as well. It was demonstrated that neutrophils express inflammatory cytokines and chemokines, and their viability is demonstrated to be affected by OSA [14].

On the other hand, OSA is tightly related to all the elements of metabolic syndrome, and obesity is the most common and best studied factor. Adipose tissue is a source of oxidative stress itself as well. It was demonstrated that neutrophils generate high amounts of ROS in obese subjects, and ROS production decreases after body mass reduction [15]. As there is no clear evidence for association of neutrophil ROS production and obesity in OSA, this study was designed aiming to clarify these possible interactions. The objective of the study was to evaluate peripheral blood neutrophil production of ROS in men with obstructive sleep apnea and establish relations with disease severity and obesity. 

## 2. Methods

The study was carried out at Pulmonology and Immunology Department of Lithuanian University of Health Sciences. It was approved by the Kaunas Regional Biomedical Research Ethics Committee (P1-48/2004, version 4, 2010). All the subjects gave their informed consent prior the study. 

Patients complaining of OSA symptoms were examined. Medical history was recorded, and physical examination was performed by a respiratory physician. None of the subjects has been previously examined or treated for sleep disorders. Inclusion criteria were male gender and age 18–60 years. Exclusion criteria were any known chronic systemic disease, chronic use of medications, infection occurring during the time of examination, alcohol abuse, and smoking. Sleepiness was evaluated using the *Epworth* sleepiness scale (ESS) [16].

All the subjects underwent a whole night attended polysomnography (PSG) using a computerized polysomnography system (*Alice 4*, Respironics). The following parameters were documented: sleep stage electroencephalogram (EEG), electrooculogram and chin miogram, electrocardiogram, airflow, thoracic and abdominal movements, oxygen saturation (SpO_2_), body position, leg movements, and snoring. Sleep recordings were scored manually. EEG was scored in 30 seconds (s) epochs and staged according to the criteria of Rechtshaffen and Kales [17]. An apnea was defined as cessation of breathing for >10 s, and a hypopnea was defined as a reduction of airflow ≥50% associated with desaturation of ≥3% or an EEG arousal, both lasting for ≥10 s [18]. An apnea/hypopnea index (AHI) was calculated as the total number of apneas and hypopneas per hour of sleep. An oxygen desaturation index (ODI) was calculated and defined as the total number of desaturations ≥3% per hour of sleep. An arousal index (AI) was calculated and defined as the total number of arousals per hour of sleep. Average oxygen saturation during sleep time (average SpO_2_) and percent of total sleep time spent with SpO_2_ < 90% (TST% SpO_2_ < 90%) were evaluated. OSA was defined as AHI ≥ 5 events per hour of sleep and daytime and nighttime symptoms as suggested in International Classification of Sleep Disorders by American Academy of Sleep Medicine [19].

Venous blood samples were obtained at 7 AM in a fasting state in the morning after the diagnostic PSG. Lipid profile consisting of total cholesterol (TC), high-density lipids (HDL), low-density lipids (LDL), triglycerides (TG), and glucose concentration were measured by standard enzymatic methods for routine estimation.

For ROS analysis, peripheral blood samples were obtained during the same morning at 10 AM into 20 mL vacutainers with ethylenediaminetetraacetic acid (EDTA). Neutrophils were isolated by density gradient centrifugation using Ficoll-Paque at 1000 g for 30 minutes at room temperature. Neutrophil population was separated using hypotonic erythrocyte lysis. Isolated neutrophils were diluted in a cell culture RPMI media to a final concentration of 2.5 × 10^6^/mL. Neutrophil ROS was determined by the formation of the fluorescent compound rhodamine-123 from dihydrorhodamine-123 (DHR-123). 60 *μ*L samples of neutrophil suspensions were incubated with 60 *μ*L DHR solution (100 *μ*g/mL) and 60 *μ*L RPMI. ROS production was measured by flow cytometer (*FACSCalibur *cytometer, Becton Dickenson, USA). For each measurement, 10^4^ events were collected. Forward and side lights showed size and granularity of the collected cells and served to determine the purity of suspensions. Data were analyzed using *CellQuest *software. Mean cellular fluorescence intensity (MFI) was calculated.

Anthropometric measurements such as height, weight, and circumferences of waist were taken during the same morning. Body mass index (BMI) was calculated by dividing weight in kilograms by squared height in meters (kg/m^2^).

All the subjects were divided into four groups: nonobese mild-to-moderate OSA (AHI < 30/hour and BMI < 30 kg/m²); obese mild-to-moderate OSA (AHI < 30/hour and BMI ≥ 30 kg/m²); nonobese severe OSA (AHI ≥ 30/hour and BMI < 30 kg/m²), obese severe OSA (AHI ≥ 30/hour and BMI ≥ 30 kg/m²). The control group was formed from men complaining of snoring and to whom OSA was rejected by the whole night PSG, when AHI was <5/hour of sleep [19].

Statistical analysis was performed using standard statistical software package *SPSS 18.0* (SPSS Inc. Chicago, IL, USA). Descriptive results for continuous variables are presented as median and 25th and 75th percentiles. Comparison between OSA patient and control groups was established using Mann-Whitney *U* test. Comparisons among the four study groups were calculated using the *Kruskal-Wallis* test. Subsequent pairwise comparisons were made with *Bonferroni* multiple comparison test. Bivariate correlations were calculated using *Spearman's* coefficient. A *P* value < 0.05 was considered to be statistically significant. 

## 3. Results

A total number of 46 patients with OSA and 10 control subjects were included in the study. Characteristics are presented in Table 1. The two groups were age matched. The study group differed from the controls in terms of all polysomnographic parameters and all measurements referring metabolic syndrome. Waist circumference was smaller and blood HDL, TG, and glucose levels were lower in the control group and did not meet diagnostic criteria of metabolic syndrome (metabolic syndrome: adult treatment panel definition III: waist circumference > 102 cm in men, TG ≥ 1.77 mmol/L, HDL < 1.0 mmol/L, and glucose ≥ 6.1 mmol/L) [20]. As expected, there was significant difference in peripheral blood neutrophil ROS production showing activation of ROS generation in OSA patient group. 

**Figure 1:**
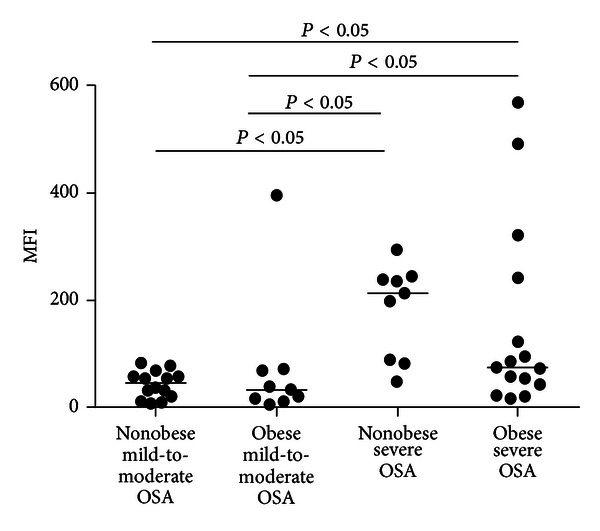
Neutrophil ROS production in obese and nonobese men with mild-to-moderate and severe obstructive sleep apnea. Data is presented as median. Significant differences between groups are marked. Comparisons of variables among four groups were made with *Kruskal-Wallis *test, followed by *Bonferroni* test. OSA: obstructive sleep apnea.

Characteristics of subjects with OSA divided into four study groups are presented in Table 2. The two groups of nonobese patients and two groups of obese patients were BMI matched, and the two groups of mild-to moderate OSA were AHI matched. Waist circumference was higher in obese patient groups compared to nonobese. The two groups of patient with severe OSA differed from mild-to-moderate OSA groups in terms of all polysomnographic parameters. Despite those differences there was no significant difference among the four groups in any blood parameter except ROS production. Differences in neutrophil ROS production are demonstrated in Figure 1. Neutrophil ROS production was higher in nonobese severe OSA group compared to nonobese mild-to-moderate OSA (MFI, median (25th and 75th percentiles) 213.4 (89.0–238.9) versus 44.5 (20.5–58.4), *P* < 0.05). In obese patient groups, ROS production was more increased in severe OSA compared to mild-to-moderate OSA group (MFI, median (25th and 75th percentiles) 74.5 (47.9–182.4) versus 31.0 (14.8–53.8), *P* < 0.05). It did not differ in the groups with different BMI and the same severity of OSA. 

Correlation coefficients between ROS production and polysomnographic parameters in OSA patients are presented in Table 3. Levels of neutrophil-generated ROS demonstrated positive correlation with AI, ODI, TST% SpO_2_ < 90% and negative correlation with Average SpO_2_. Neither BMI (*r* = 0.074, *P* > 0.05) nor waist circumference (*r* = −0.038, *P* > 0.05) was significantly correlated with neutrophil ROS. No significant relation was detected between ROS production and any parameter of lipid profile as well.

## 4. Discussion

Differences in peripheral blood neutrophil ROS production in men with OSA were evaluated in this study. The design of the study was planned trying to control potential confounding factors such as gender, age, smoking, and other comorbidities that were reported to be possibly responsible for the discrepancies among different studies evaluating oxidative stress [21]. All the groups were age matched referring to middle-aged population. All the subjects were nonsmokers. Trying to avoid gender differences only men were included in the study. Male predominance in OSA is well known, but the reasons remain unclear. Possible explanations include hormonal effect on upper airway collapsibility, differences in body fat distribution as well as pharyngeal anatomy [22]. The other important point is that all the subjects were healthy except increased weight and OSA. Even lipid concentrations and fasting glucose levels did not exceed normal ranges. 

The study groups of obese and nonobese patients were formed choosing the cut point of 30 kg/m^2^. BMI ≥ 30 kg/m^2^ was considered obesity. Unfortunately we did not succeed to avoid overweight subjects in nonobese patient groups. The control group served in distinguishing OSA patients that were affected by intermittent hypoxia during sleep from the healthy ones, and this was proved by performing polysomnography—a gold standard procedure in diagnostics of OSA. The control group was healthy in all terms of metabolic syndrome as well. To our minds such an accurate selection of study participants is the strong part of this study. 

Obesity itself is proved to be an important factor for developing and progression of OSA. Both Wisconsin Sleep Cohort study and the Sleep Heart Health Study have shown the impact of changes in body weight on the natural course of OSA [23, 24]. On the other hand, obesity is demonstrated to be a factor of increased oxidative stress as well [25]. On the ground of this four study groups were formed. 

Controversial data could be found in literature on production of neutrophil ROS in OSA [7, 26]. In the study by Muns and coworkers, neutrophil oxidative burst in blood neutrophils of 24 OSA patients was evaluated [26]. Radiolabeled dihydrorhodamine was used to detect oxidative burst elicited by incorporation of *Escherichia coli.* Oxidative burst appeared not to be increased in OSA group compared with healthy controls [26]. Schulz and his colleagues developed this idea and challenged neutrophils with nonbacterial stimuli [7]. They formed the study group of 18 men with OSA (mean AHI 53 ± 6 events/h) and compared it to healthy controls. Markedly enhanced readiness of neutrophils to respond with superoxide generation to different stimuli was found in patients with OSA. Furthermore, it was rapidly reversible with continuous positive airway pressure (CPAP) therapy [7]. Unfortunately a trial of treatment with CPAP was not planned in our study. It would be very interesting to have such results as the group of Schults had demonstrated changes in superoxide release from polymorphonuclear neutrophils already after two nights of CPAP treatment [7].

The most interesting finding of the study is that increased neutrophil ROS production was related to severity of OSA. In the groups with severe OSA more fragmented sleep, greater level of nocturnal hypoxemia and intermittent hypoxia and more increased ROS production was found. There was no such relation with anthropometric parameters as BMI and waist circumference. One should pay attention that there was no statistical significance in BMI between the two groups of severe OSA though these groups differed in AHI. Actually different results could be expected, as recent study has demonstrated that OSA was related to central obesity and not to intermittent hypoxia [27]. The explanation supporting our results could be the precise selection of patients without any confounders. 

## 5. Conclusions

Neutrophil ROS production was increased in severe OSA groups compared to mild-to-moderate OSA, but did not differ in obese and nonobese patient groups with the same severity of OSA. Increased neutrophil ROS production was related to more severe OSA but not obesity.

The results of this study support the hypothesis that increased ROS production in peripheral blood neutrophils is provoked by intermittent hypoxia and may activate inflammatory cascade leading to the development of impaired endothelial function and contribute to the development of cardiovascular diseases. Further studies showing the effect of CPAP treatment on neutrophil ROS production in obese and nonobese OSA patients could support these findings. 

## Figures and Tables

**Table 1:** Characteristics of the controls and the obstructive sleep apnea patients.

Characteristic	Control group (*n* = 10)	OSA group (*n* = 46)	*P* value
Age (years)	44 (32–46)	43 (35–50)	>0.05
*Epworth *sleepiness scale	9 (9–13)	5.5 (5–11)	0.39
Body mass index (kg/m^2^)	25.3 (23.7–27.8)	30.6 (28.4–36.6)	**<0.001**
Waist circumference (cm)	90 (87–93)	106 (98–116)	**<0.001**
Polysomnographic parameters:			
Apnea/hypopnea index (events/h)	2.6 (2.1–3.4)	29.9 (15.8–70.2)	**<0.001**
Arousal index (events/h)	19.1 (14.2–24.1)	39.3 (21.6–58.4)	**0.001**
Oxygen desaturation index (events/h)	2.9 (1.0–3.8)	42.9 (12.8–74.2)	**<0.001**
Average SpO_2_ (%)	97 (96-97)	94 (92–95)	**<0.001**
Total sleep time SpO_2_ < 90% (%)	0 (0–0.2)	4.4 (0.4–24.3)	**<0.001**
Blood parameters:			
Total cholesterol (mmol/L)	4.99 (4.88–5.62)	5.68 (4.94–6.48)	>0.05
High-density lipids (mmol/L)	1.16 (1.02–1.51)	0.91 (0.79–1.04)	**0.01**
Low-density lipids (mmol/L)	3.24 (3.07–3.61)	3.27 (3.15–4.21)	>0.05
Triglycerides (mmol/L)	0.92 (0.79–1.11)	1.73 (0.94–2.57)	**0.03**
Glucose (mmol/L)	5.0 (4.29–5.33)	5.59 (5.31–5.93)	**0.01**
ROS (MFI)	28.29 (16.5–58.1)	58.44 (29.9–108.4)	**0.02**

Data is presented as median (25th and 75th percentiles). Mann-Whitney *U* test. Statistically significant differences are bolded.

**Table 2:** Characteristics of obese and nonobese men with mild-to-moderate and severe obstructive sleep apnea.

Variable	Nonobese mild-to-moderate OSA (*n* = 14)	Obese mild-to-moderate OSA (*n* = 8)	Nonobese severe OSA (*n* = 9)	Obese severe OSA (*n* = 15)
Age (yearns)	45.5 (41–48)	43.0 (39–46)	48.0 (32–54)	37.0 (34–48)
*Epworth *Sleepiness Scale	10.5 (4.0–12.0)	10.0 (5.5–12.5)	10.0 (4.0–15.0)	11.0 (10.0–17.0)
BMI (kg/m^2^)	28.2 (28.1–28.7)^†#^	34.1 (31.3–38.4)^∗¥^	28.4 (27.5–29.8)^†#^	37.4 (33.8–38.7)^∗¥^
Waist (cm)	100 (95–102)^†#^	115 (110–118)^∗¥^	97 (96–101)^†#^	118 (112–126)^∗¥^
Polysomnographic parameters:				
AHI (events/h)	12.7 (8.5–19.9)^¥#^	16.2 (9.1–19.4)^¥#^	53.5 (35.5–59.4)^∗†#^	65.3 (74.2–91.4)^∗†¥^
Arousal index (events/h)	21.7 (18.9–27.5)^¥#^	19.9 (14.5–24.3)^¥#^	38.2 (33.4–59.4)^∗†#^	59.1 (48.5–79.9)^∗†¥^
Oxygen desaturation index (events/h)	11.5 (7.1–13.7)^¥#^	15.6 (9.9–18.9)^¥#^	48.8 (27.1–56.3)^∗†#^	76.1 (72.2–78.9)^∗†¥^
Average SpO_2_ (%)	96.0 (94.0–96.0)^#^	94.5 (94.0–95.5)^#^	95.0 (93.0–95.0)^#^	91.0 (87.5–92.0)^∗†¥^
Total sleep time SpO_2_ < 90% (%)	0.1 (0–1.4)^#^	0.8 (0.3–2.0)^#^	5.3 (2.5–7.2)^#^	34.5 (23.2–53.2)^∗†¥^
Lipid profile:				
Total cholesterol (mmol/L)	5.90 (4.92–6.64)	5.64 (5.29–6.69)	5.46 (4.91–6.07)	5.15 (4.93–6.24)
High-density lipids (mmol/L)	1.01 (0.96–1.05)	0.76 (0.70–0.86)	0.95 (0.81–1.20)	0.91 (0.80–1.02)
Low-density lipids (mmol/L)	3.84 (3.37–4.86)	3.74 (3.61–4.51)	3.22 (3.05–3.71)	3.75 (3.32–3.94)
Triglycerides (mmol/L)	1.14 (0.86–2.41)	2.06 (1.84–2.67)	1.22 (0.85–2.56)	1.70 (1.19–2.92)

Data is presented as median (25th and 75th percentiles). Comparisons of variables among four groups were made with *Kruskal-Wallis *test, followed by *Bonferroni* test. OSA: obstructive sleep apnea. *P < 0.05 versus nonobese mild-to-moderate OSA, ^†^ *P* < 0.05 versus obese mild-to-moderate OSA, ^¥^ *P* < 0.05 versus nonobese severe OSA, ^#^ *P* < 0.05 versus obese severe OSA.

**Table 3:** Correlation coefficients between neutrophil reactive oxygen species and polysomnographic parameters in obstructive sleep apnea patients.

Variable	*r*	*P*
Apnea/hypopnea index (events/h)	0.429	**0.003**
Arousal index (events/h)	0.502	**<0.001**
Oxygen desaturation index (events/h)	0.295	**0.047**
Average SpO_2_ (%)	−0.310	**0.036**
Total sleep time SpO_2_ < 90% (%)	0.337	**0.022**

*Spearman's rho* coefficient. Statistically significant differences are bolded.
